# Genomic comparison of methicillin-resistant *Staphylococcus aureus* CC398 isolates from livestock, meat and humans in the Netherlands

**DOI:** 10.3389/fmicb.2026.1770695

**Published:** 2026-02-09

**Authors:** Engeline van Duijkeren, Cindy M. Dierikx, Michael S. M. Brouwer, Kees T. Veldman, Bart Wullings, Michel Rapallini, Ben Wit, Tryntsje Cuperus, Paul D. Hengeveld, Angela H. A. M. van Hoek, Angela de Haan, Sandra Witteveen, Antoni P. A. Hendrickx

**Affiliations:** 1 National Institute for Public Health and the Environment (RIVM), Centre for Infectious Disease Control, Bilthoven, Netherlands; 2 Wageningen Bioveterinary Research (WBVR), Lelystad, Netherlands; 3 Wageningen Food Safety Research (WFSR), Team Bacteriology, Molecular Biology and AMR, Wageningen, Netherlands; 4 Netherlands Food and Consumer Product Safety Authority (NVWA), Food Safety, Utrecht, Netherlands

**Keywords:** antimicrobial resistance, CC398, GG0398, LA-MRSA, wgMLST

## Abstract

Since 2003, methicillin-resistant *Staphylococcus aureus* clonal complex 398 (MRSA- CC398) emerged in livestock. To assess possible transfer of resistant strains, animal-related MRSA- CC398 were characterized and compared to those from humans. For that purpose, MRSA-CC398 isolates (*n* = 2,569) from the national human MRSA surveillance (*n* = 1,758) and animal-related isolates (*n* = 811) were included for analysis. Next-generation sequencing data were used for MLST, whole-genome MLST (wgMLST) and identification of resistance/virulence genes. wgMLST showed that animal- and human-related MRSA-CC398 isolates grouped together in four groups termed A (*n* = 205), B (*n* = 308), C (*n* = 382), and D (*n* = 1,674) varying 103–139 wgMLST alleles between groups. Some animal-related isolates were closely related to human isolates or to animal isolates from other farms in all four groups. There were no groups containing animal isolates only. Differences were identified in the prevalence of virulence- and resistance genes between MRSA-CC398 originating from human- and animal-related isolates and between the four groups. Specifically, one branch within group C comprised only MRSA-CC398 from humans; these isolates were often ST1232, and carried *lukS-PV/lukF-PV* (PVL)-, *sak, scn, ant*(*9*)-Ia, and *erm*(A)-variant genes. Persons carrying this lineage rarely reported professional livestock contact, lived in areas with low pig density, and had more often a clinical infection compared to persons carrying non-ST1232. The MRSA-CC398 population in the Netherlands is diverse and comprised of four groups with distinct genomic signature. Although most MRSA-CC398 are still livestock-associated, a PVL-positive human-related lineage in CC398 has emerged in the Netherlands among the population in the absence of livestock contact.

## Introduction

The Netherlands is a country with a low endemic level of methicillin-resistant *Staphylococcus aureus* (MRSA) in humans due to the restricted use of antibiotics and implementation of a so-called “Search and Destroy” policy. This policy includes active screening of high-risk groups upon hospital admission, preventive isolation, and treatment of MRSA carriers (Vandenbroucke-Grauls, 1996; Wertheim et al., 2004). During the last decades, livestock has emerged as an important source of MRSA, colonizing and infecting humans worldwide (Cuny et al., 2015; Cui et al., 2025). The epidemiology of livestock-associated MRSA (LA-MRSA) differs among geographic regions. In Europe, MRSA clonal complex (CC) 398 is the predominant genomic group found in livestock (European Food Safety Authority and European Centre for Disease Prevention and Control, 2025). In 2023, MRSA-CC398 made up 13% of all isolates submitted in the Dutch human MRSA surveillance program (de Greeff, 2024). The most important risk factor for carriage and infections with MRSA-CC398 in humans is professional contact with livestock. A recent meta-analysis concluded that livestock-exposed populations were 10-fold more likely to be colonized by LA-MRSA than the control population (Chen and Wu, 2020). In particular, pig farmers had the highest odds ratio (15.4) for LA-MRSA colonization compared to the control group (Chen and Wu, 2020). During the last decade, however, the number of persons colonized or infected with MRSA-CC398 in the Netherlands who did not have professional contact with livestock, is increasing (van Rijen et al., 2014; Schouls et al., 2023) and a similar observation was made in Denmark (Larsen et al., 2015). Two Dutch studies found that, respectively, 21 and 76% of persons carrying or infected with MRSA-CC398 did not report direct contact with pigs, broilers or veal calves (van Rijen et al., 2014; Konstantinovski et al., 2022). In addition, long-term carriage of MRSA-CC398 in persons without professional livestock contact has been reported in a livestock-dense area (Meijs et al., 2020). It was also shown that there was frequent transmission of MRSA-CC398 between veterinarians and their household members and that both veterinarians and their household members carried the same MRSA strain for prolonged periods of time (Bosch et al., 2015), indicating within household human-human transmission.

In addition, Panton–Valentine leucocidin (PVL) positive MRSA-CC398 were increasingly found (up to 6%) in the Dutch MRSA surveillance in the years 2017–2019 with isolates predominantly found in regions with few pig farms (Schouls et al., 2023). PVL is a cytotoxin associated with skin- and soft tissue infections (Shallcross et al., 2013). As the molecular epidemiology of MRSA-CC398 is changing, there is a need for increased genomic surveillance in humans as well as animals. To date, genomic studies on/including MRSA-CC398 focused on human isolates only (Konstantinovski et al., 2022; Schouls et al., 2023), animal-derived isolates only (Hansen et al., 2019; Silva et al., 2024), analyzed a limited number of isolates (Lee et al., 2023; Abdullahi et al., 2024), or mainly used publicly available genomic data with limited metadata and underrepresentation of countries that do not routinely use next-generation sequencing (Fernandez et al., 2024). The objective of the present study was to assess the genetic relatedness and to compare the resistome and virulence factors among a large collection of MRSA-CC398 isolates originating from humans, livestock and meat in the Netherlands in the period 2001–2022. For this One Health genomic MRSA surveillance, 2,569 isolates from the national MRSA surveillance in livestock, meat, persons working/living on farms (van Duijkeren et al., 2025), livestock-related isolates from previous studies and a collection of isolates from the national MRSA surveillance in humans were analyzed. This is the largest study combining a long-term human-animal-food MRSA dataset in the Netherlands and will provide insight in the population structure as well as the dynamics of emerging lineages, resistance determinants and virulence genes in animals and humans.

## Materials and methods

### Bacterial isolates

For the Dutch national MRSA surveillance, medical microbiology laboratories (MMLs) in the Netherlands are requested to send one MRSA isolate per person colonized or infected with MRSA per 3 years to the National Institute for Public Health and the Environment (RIVM), of which ~10% are sequenced with next-generation sequencing (NGS, *vide infra*). Animal-related and isolates from meat were collected in the national surveillance by, respectively, Wageningen Bioveterinary Research (WBVR) and Wageningen Food Safety Research (WFSR). Isolates from milk of cows with mastitis were collected by Royal GD. In total, NGS data of 2,569 MRSA isolates (2016–2022) belonging to CC398 from humans (*n* = 1,758) and 811 animal-related MRSA isolates belonging to CC398 from different studies between 2001 and 2022 were included. Twenty-eight MRSA-CC398 isolated from nasal swabs from persons living and/or working on livestock farms were defined as “animal-related” isolates, as those are assumed to represent “true” livestock-associated MRSA and were sampled as part of the national surveillance of livestock and meat, whereas isolates from persons with self-reported livestock contact collected by the human national surveillance were considered to be “human” isolates, as they did not always have professional contact with animals and might have been colonized/infected through other routes.

### Metadata

MMLs provided the sampling date, the nature of the specimen, the type of health care provider, gender, age in years, four digits of the zip code and a pseudonymized person identifier. Since the introduction of the digital data exchange system, Type-Ned for MRSA surveillance, in November 2016, MMLs and infection prevention workers were requested to fill in digital questionnaires to provide additional data on persons and to assess risks factors associated with MRSA infection and colonization (Schouls et al., 2023). The questionnaires contained questions on the health care provider, the residential stay and risk factors for MRSA carriage such as underlying disease, having been and/or nursed abroad, and animal contact. Isolates were classified as screening samples when they were cultured from swabs of the nose, throat and/or perineum. For the animal-related samples the sample date, nature of the specimen and animal species was known.

### NGS and wgMLST

MRSA isolates (*n* = 2,569) were sequenced using Illumina platforms and *de novo* assembled using SPAdes 3.15.3 and CLC Genomics Workbench v20.03. For comparative analysis, the whole-genome multi-locus sequence typing (wgMLST) *S. aureus* scheme comprising 2,567 genes was used (Leopold et al., 2014). The wgMLST scheme consists of the MLST+ scheme (1,861 targets, version 1.3) and the accessory scheme (706 targets, version 1.2). For classical MLST the SeqSphere software version 6.0.2 (Ridom GmbH, Münster, Germany) was used. Both wgMLST and MLST profiles were imported into BioNumerics version 8.1.1 (Applied Maths, Sint-Martens-Latem, Belgium) and used in categorical clustering. Groups of isolates with less than 16 wgMLST alleles differences were considered as one cluster (Schurch et al., 2018). Resistance genes, SCC*mec* types and virulence determinants were identified using AMRFinder from the National Center for Biotechnology Information,^1^ and the SCC*mec*Finder and VirulenceFinder databases from the Center for Genomic Epidemiology (downloaded on 01-04-2020),^2^ respectively. A threshold of 95% was used for identity and 60% for the minimum length for detection of resistance genes using AMRFinder v3.11.11. Raw sequencing data of all Dutch MRSA surveillance isolates (2016–2022) have been deposited in the SRA database under various BioProjects (Supplementary file 1) and the animal-related isolates have been deposited in BioProject PRJEB83095.

### Ethics statement

The bacterial isolates belong to the MMLs participating in the National MRSA surveillance program and were obtained as part of routine clinical care in the past years. Since no identifiable personal data were collected and data were analyzed and processed anonymously, written or verbal patient consent was not required. For the isolates from the persons working/living on the farms, participants signed informed consent forms. All personal data were processed in line with the General Data Protection Regulation. According to the Dutch Medical Research Involving Human Subjects Act (WMO) the study was exempt from review by an Institutional Review Board. All procedures for the sampling of animals were in accordance with ethical standards of the Dutch Law on Animal Health and Welfare.

## Results

### Bacterial isolates

The MRSA-CC398 isolates (*n* = 811) collected at farms and slaughterhouses originated from Dutch broilers, dairy cattle, veal calves, ducks, turkeys, pigs, dust collected in the sheds/barns/broiler houses of farms or at the slaughterhouse, Dutch retail meat, and from nasal swabs from persons living and/or working on livestock farms (Table 1). In the studies included, different sample types (dust, nasal swabs, skin swabs) were used for the different farm animals. This was done because the best sample type varies per animal species, the objective of the studies included was not the same or because of practical reasons. These isolates were defined as “animal-related” isolates, as those are assumed to represent “true” livestock-associated MRSA. Four-hundred sixty animal-related isolates were from the period 2016–2022, while 351 isolates were from the period 2001–2015. Most isolates originated from (the environment of) apparently healthy animals or meat thereof, except for the 89 milk samples which originated from cows with (sub) clinical mastitis.

**Table 1:** Origin of the animal-related MRSA isolates.

Animal species	Human*	Farm	Retail	Slaughter-house	Animal				Total
	Nose swab	Dust	Meat	Dust	Feces	Throat/nose swab	Skin	Milk**	
Broiler	13	3	36	10	3	16			81
Cattle	1	17	33	1	1		28	89	170
Duck		1				1			2
Pig		117	15		26	135			293
Turkey	7	9	4			3			23
Veal calves	7		9		110	116			242
Total	28	147	97	11	140	271	28	89	811

*Persons living/working on a farm. **Isolated from milk from cows with mastitis.

The 1,758 MRSA-CC398 isolates from the human MRSA surveillance originated from 1,100 (62.6%) males and 658 (37.4%) females aged between 0 and 102 years, median 54 years, 25% percentile 32 years, 75% percentile 67 years; 528 (30.2%) were obtained from clinical cases, 1,223 (69.8%) originated from screening swabs and for 7 isolates this information was unknown. These isolates were defined as human-related isolates. Professional contact with livestock was reported for 677/1,005 persons (67.4%), 328/1,005 (32.6%) had no professional contact with livestock, and in 753 cases this information was unknown or not recorded. Most MRSA-carrying persons lived in provinces of the Netherlands with high pig density such as Noord-Brabant (*n* = 759), Gelderland (*n* = 339), Overijssel (*n* = 195) and Limburg (*n* = 153), while few MRSA-CC398-positive persons lived in provinces with low pig density such as Zuid-Holland (*n* = 57); Noord-Holland (*n* = 58); Utrecht (*n* = 50); Drenthe (*n* = 44); Groningen (*n* = 28); Zeeland (*n* = 22); Flevoland (*n* = 19), and Friesland (*n* = 18); for 16 persons this information was not recorded. Seventeen persons had been abroad during the past 6 months, 593 persons had not been abroad, and for 1,148 persons this information was not recorded. In 9/17 cases persons had traveled to other European countries, in 1/17 cases they travelled to Turkey, in 4/17 cases to Vietnam and in 1/17 cases to Thailand.

All isolates carried the *mecA* gene and *mecC-*positive MRSA*-*CC398 were not detected in this dataset. SCC*mec* type IV was found in 17.9% of the human-related MRSA-CC398 isolates and 40.2% of the animal-related MRSA-CC398 isolates. SCC*mec* type V was found in 81.6% of the human-related MRSA isolates and 58.0% of the animal-related isolates. SCC*mec* type Vb (5C2&5) was the most prevalent type in both human- and animal-related isolates, while SCC*mec* type IVa(2B) was the second most common type (Table 2).

**Table 2:** Distribution of the SCC*mec* types among human-related and animal-related MRSA CC398.

Species	Human-related	Animal-related Total	Broiler-related	Turkey-related	Pig-related	Veal calf-related	Cattle-related
*N*	*n* = 1,758	*n* = 811	*n* = 81	*n* = 23	*n* = 293	*n* = 242	*n* = 170
SCC*mec type*	(%)	(%)	(%)	(%)	(%)	(%)	(%)
IV(2B&5)	0.2	a	a	a	a	a	a
IV(2B)	0.1	a	a	a	a	a	a
IVa(2B)	17.4	39.3	18.5	56.5	18.4	69.8	40.0
IVc(2B)	0.2	0.9	a	a	a	2.9	a
V(5C2)	9.8	1.7	8.6	a	2.4	a	a
Vb(5C2&5)	71.8	56.2	65.4	43.5	78.2	26.4	57.6
Other	0.5	2.0	7.4	a	1.0	1.2	2.4

a, absent.

### Genomic comparison of MRSA-CC398 subgroups A, B, C, and D

**Figure 1:**
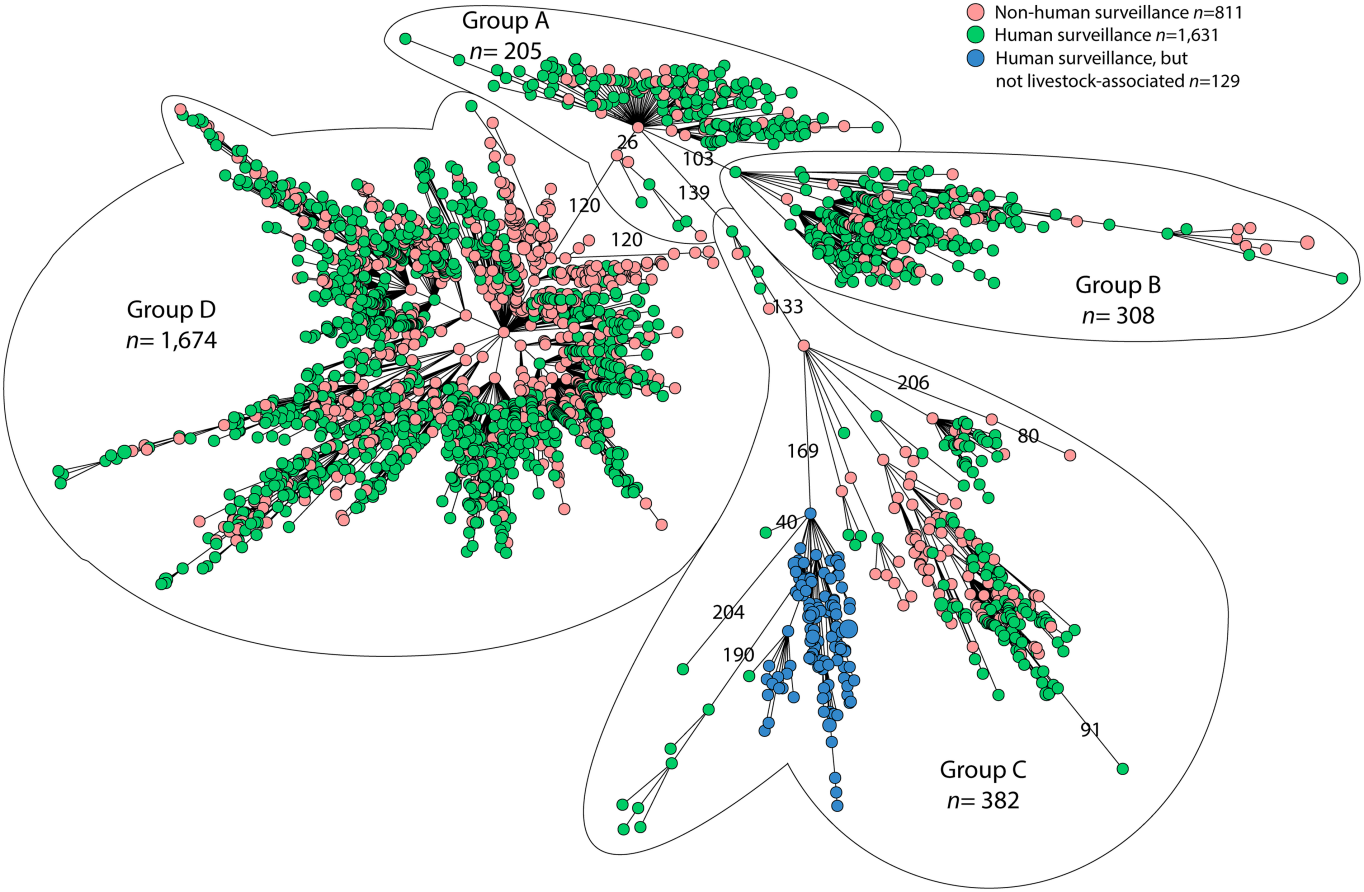
WgMLST minimum spanning tree of the human- and animal-related MRSA CC398 isolates in the Netherlands; distribution of the isolates showing four different groups CC398-A, -B, -C, and -D. Groups within CC398 differ at least 100 wgMLST alleles.

**Figure 2:**
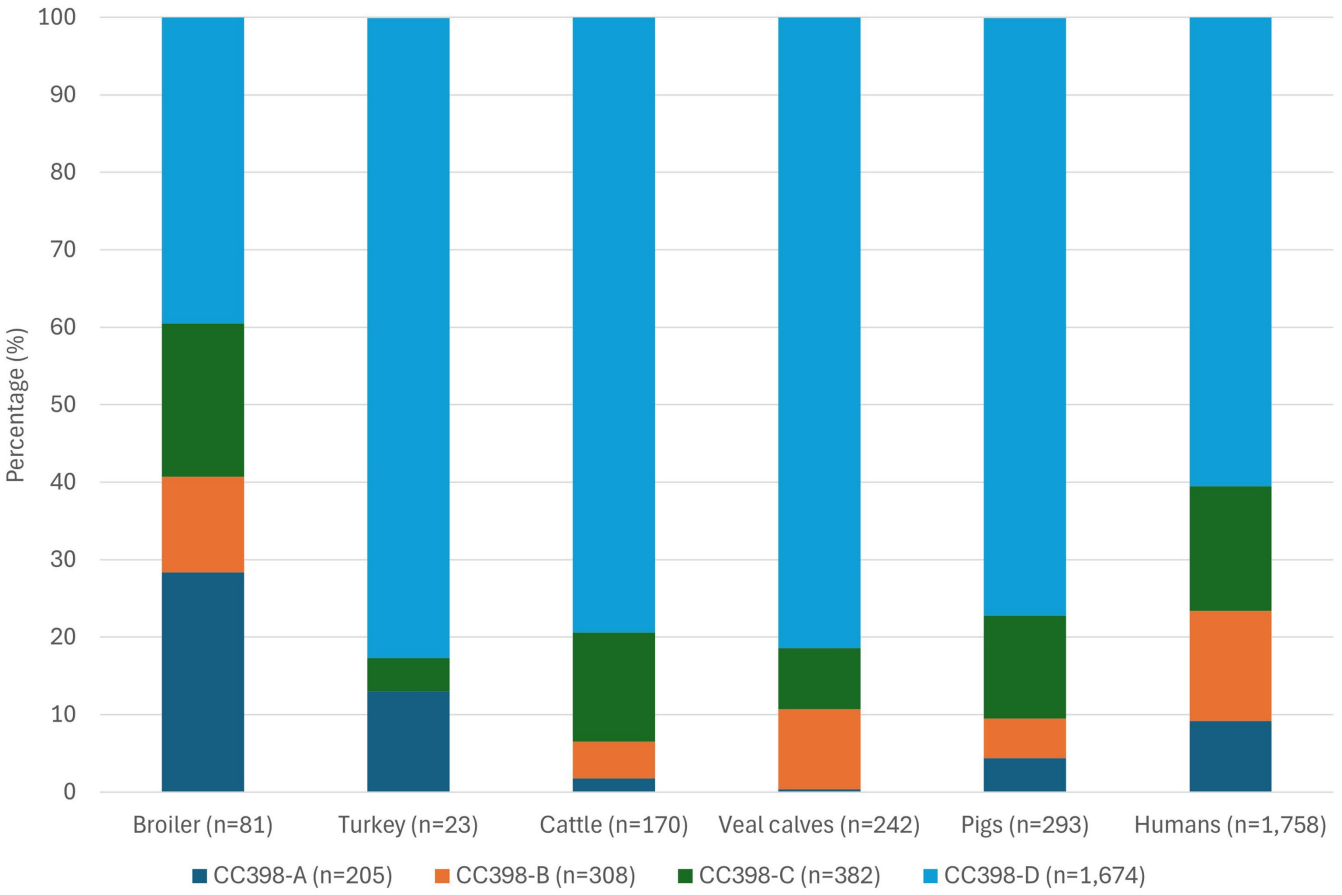
Distribution of MRSA isolates over the CC398-A–D groups originating from different animal species and humans.

Analysis of MLST types showed that 61 sequence types (ST) were found among the 2,569 MRSA-CC398 isolates, with ST398 MRSA (*n* = 2,350; 91.5%) dominating. MRSA ST1232 (*n* = 117) was the second most found ST and ST4131, ST4340 and ST7015 (*n* = 6 each) were the third common ones. Other STs were found three times or less. wgMLST analysis revealed that most animal-related MRSA-CC398 isolates grouped together with human-related MRSA isolates in the minimum spanning tree (Figure 1). Four distinct groups in CC398 could be discerned: group A comprising 205 isolates, group B consisting of 308 isolates, group C containing 382 isolates and the largest group D consisted of 1,674 isolates, varying by 103–139 wgMLST allelic differences between groups. All MRSA groups within the minimum spanning tree contained animal-related isolates as well as human-related MRSA isolates. However, within the diverse group C, there was one distinct branch containing human-retrieved MRSA isolates only. This branch consisted of 129 isolates: PVL-positive (*n* = 114) and PVL-negative (*n* = 3) ST1232 isolates, and the PVL-positive STs ST4081 (*n* = 1), ST4082 (*n* = 1), ST4131 (*n* = 6), ST5074 (*n* = 2), ST7646 (*n* = 1) and ST8355 (*n* = 1). ST1232 and ST4081 are single locus variants of ST398, whereas the other STs are double locus variants of ST398. Persons with this human MRSA-CC398 lineage had limited professional livestock contact (1/47; 2.1%; in 82 cases this information was unknown) compared to persons outside this branch (573/827; 69.3%; in 77 cases this was unknown) and had more often a clinical infection (82/129; 63.6%) compared to persons outside this lineage (403/1,552; 26.0%; for 77 persons this was unknown). Most persons infected or colonized with this human lineage lived in areas with low pig density such as Noord-Holland (*n* = 29) and Zuid-Holland (*n* = 16) compared to the other types of MRSA-CC398. Nearly all isolates within the human lineage carried subtype 5C2 of SCC*mec* type V (97.7%). Figure 2 shows that the isolates from all animal species belong to the four different CC398 subgroups A to D, except for turkey isolates, which were not found in MRSA CC398-B.

### Genetic clusters of MRSA-CC398

Within the four groups of MRSA-CC398, multiple types of genetic clusters could be identified with isolates varying less than 16 wgMLST allelic differences.

#### Human-animal genetic clusters

In total, there were 62 human-animal MRSA-CC398 clusters comprising 320 isolates: 181 human-related isolates and 139 animal-related isolates. The smallest clusters (*n* = 19) consisted of only 2 isolates, while the largest contained 28 isolates. The majority of the animal-related isolates within the clusters originated from pigs: pig-related isolates (*n* = 85) were found in 49 clusters. Cattle-related isolates were second most commonly found in genetic clusters: in 17 clusters with 31 isolates. In CC398-A, there were seven human-animal MRSA clusters, in CC398-B five, in CC398-C four, and in CC398-D 46. Thirteen clusters overlapped in sampling year and geographic location (on Province level). For fifteen clusters, geographical location of the animal isolates was not known.

#### Farmer-animal cluster

There were six clusters containing in total 22 isolates: fifteen from animals, six from farmers and one from a family member of a farmer. In four clusters the animal (all from poultry) and human isolates within each cluster originated from the same postal code, indicating that they were living on the same farm. In one cluster the isolates were from a cow and a dairy farmer from different provinces of the Netherlands without known epidemiological link, except that both isolates were from 2021. The largest cluster contained six isolates, the isolates were from two pigs isolated in 2015 at the same slaughterhouse (but originating from different farms) in the province of Gelderland, while three turkey isolates and a farmer isolate were collected at the same farm in the province of Overijssel in 2014. Based on the results of the questionnaire no epidemiological link was found between the pig isolates and the turkey/turkey farmer isolates.

#### Farmer-human surveillance cluster

There were two genetic clusters containing a screening isolate from a veal calf farmer and a MRSA isolate obtained from the national human surveillance. The two pairs of isolates were not related in time of sampling nor regarding the home address as based on postal code. Both isolates from the national MRSA surveillance derived from persons having animal contact (pigs and cattle respectively).

#### Animal–animal clusters

In total, there were 72 clusters containing only animal-related isolates (*n* = 187). Most clusters contained isolates from broilers/broiler meat/broiler dust (*n* = 9), pigs/pork/pig dust (*n* = 21) or veal calves/veal (*n* = 20) only, but there were also mixed clusters (*n* = 9) containing isolates from two or more animal species, such as pig and broiler (*n* = 1), pig and veal calf (*n* = 2) or pig and cattle (*n* = 6). In 18/72 cases an epidemiological link could be found as the samples were taken at the same slaughterhouse or originated from the same farm. For the other 54 clusters no epidemiological link was found.

### Virulence factors among MRSA-CC398 groups A, B, C, and D

All human- and animal-related MRSA-CC398 isolates from groups A-D carried the haemolysin genes *hlgA*, *hlgB*, and *hlgC*. The *lukF*-*PV* and *lukS-PV* genes, encoding PVL, were found in 127/1,758 (7.2%) human isolates. All but one of these isolates belonged to the previously described human lineage (mostly ST1232) in group C and only one PVL-positive human CC398 isolate was found outside this human lineage in group D. None of the animal-related isolates from groups A, B, C, and D were PVL-positive. Other genes encoding for virulence factors were found only sporadically in human isolates, including the *lukD* and *lukE* leucocidins in two isolates (one in group C and one in group D), the toxic shock syndrome gene *tst* in one isolate (group A), the serine protease genes *splA*, *splB* in two isolates (one in group C and one in group D), and the serine protease gene *splE* in one isolate (group C). The staphylokinase gene *sak* was found in 213 (12.1%) human isolates (group A/B/C/D) and in 8/811 (1.0%) animal-related isolates (group B and D), whereas staphylococcal complement inhibitor gene *scn* was found in 217 (12.3%) human isolates (within all groups) and nine (1.1%) animal-related isolates (group B and D). The *sak* and *scn* positive animal-related isolates were broiler related (*n* = 5) and from cattle (*n* = 3). The staphylococcal enterotoxin B gene *seb* was found in 62 (3.5%) isolates from humans and in 13 (1.6%) animal-related isolates, of which nine originated from pigs. Virulence genes encoding for other enterotoxins (*sea, sec, sed, sej, sek, sel, sep, seq,* and *ser*) were rarely found (Table 3; Supplementary Table S1).

**Table 3:** Distribution of virulence factors among MRSA-CC398 isolates from humans, isolates belonging to the human lineage (group C) and animal-related isolates (group A, B, C, D).

Virulence gene category	Species	Human total	Human lineage	Animal-related
	Number	*n* = 1,758	*n* = 129	*n* = 811
	Virulence genes	(%)	(%)	(%)
Host immunity	*sak*	12.1	99.2	1.0
	*scn*	12.3	99.2	1.1
Toxin	*lukF-PV*	7.2	97.7	a
	*lukS-PV*	7.2	97.7	a
	*tst*	0.06	a	a
Enterotoxin	*seb*	3.5	0.0	1.6

a, absent.

### Antimicrobial resistance among MRSA-CC398 groups A, B, C, and D

The distribution of predicted antimicrobial resistance genes among MRSA-CC398 isolates was assessed. The number of resistance genes (*n*/N*100%) and the type of genes predicted to be implicated in resistance differed between MRSA isolates from humans, isolates in the human lineage and isolates from animals. In addition, there were also differences in the prevalence of several resistance genes between isolates originating from different animal species (Supplementary Table S2). All isolates from the human ST1232 lineage within group C carried *ant*(*9*)-*Ia* (also called *spc*) encoding resistance to spectinomycin and a variant of *ermA* encoding resistance to macrolides, lincosamides and streptogramin B (MLS_B_ resistance). The *ermA*-variant was identified by ResFinder, as AMRFinder was not able to detect this variant of *ermA* (Brodikova et al., 2025). Ninety-three percent carried the tetracycline resistance gene *tet*(K) and none of the isolates carried *tet*(M). Resistance genes predicted to encode resistance to trimethoprim or linezolid were absent and resistance genes encoding resistance to phenicols were only rarely found in this human lineage. Genes implicated in linezolid-resistance such as *optrA,* and *cfr* were rare in the whole dataset.

Almost all (>97%) human and animal-related isolates in group A carried *ant*(*9*)*-Ia*, encoding spectinomycin resistance, *vga*(E) encoding pleuromutilin, lincosamides, streptogramin A resistance, *erm*(A) encoding macrolide resistance, *dfrG* encoding trimethoprim resistance and *tet*(M) encoding tetracycline resistance, while most of these genes were very rarely (<3% of all isolates) found in isolates belonging to group D (Supplementary Table S2). Mapping of five randomly chosen isolates from group A showed that the resistance genes *ant(9)-Ia*, *vga*(E), and *erm*(A) were located on transposon Tn6113 as described by Schwendener and Perreten (2011). This makes these strains multi-resistant by carrying only one mobile genetic element. In addition, there was one branch within group D with predominantly veal calf isolates (*n* = 197; 81.4% of all isolates from veal calves). These veal calf isolates in group D often carried aminoglycoside resistance genes *aac(6′)-Ie- aph(2″)-Ia* (*n* = 164/197; 83.2%), *aadD1* (*n* = 122/197; 61.9%) and the MLS_B_ resistance gene *erm*(T) (*n* = 127/197; 64.5%), while these genes were less common in isolates from veal calves outside group D *aac(6′)-Ie- aph(2″)-Ia* (*n* = 4/45; 8.9%), *aadD1* (*n* = 18/45; 40.0%), and *erm*(T) (*n* = 1/45; 2.2%).

## Discussion

MRSA-CC398 is genetically diverse and widely disseminated in livestock and humans in the Netherlands. wgMLST analyses showed that MRSA-CC398 isolates originating from animals, dust from the barns and slaughterhouses, meat and humans in the Netherlands generally grouped together but could be divided into four major groups CC398-A, -B, -C, and -D. These groups varied by at least 100 wgMLST allelic differences and all contained human as well as animal-related isolates. Sixty-two genetic clusters were found with isolates from livestock and humans differing by less than 16 allelic differences, suggesting dissemination of MRSA between livestock and humans. It should be noted, however, that wgMLST similarity alone does not prove direct transmission and that unsampled reservoirs or indirect transmission routes cannot be excluded. There were also clusters of isolates differing less than 16 allelic differences originating from animals only and these clusters sometimes contained isolates from different farms and from multiple animal species, indicating dissemination of MRSA-CC398 between farms and different livestock sectors of which the source, however, remains unknown.

A limitation of the current study concerns the temporal heterogeneity of the collection: the animal-related isolates were collected between 2001 and 2022, whereas human MRSA surveillance isolates were from 2016 to 2022. In addition, limited metadata were available for the animal-related isolates and metadata were sometimes incomplete for the human MRSA surveillance isolates. The expression of resistance genes found was not confirmed by phenotypic susceptibility testing. Direct epidemiological linkage data was not always available due to restriction/regulations regarding the privacy of patients and farmers/livestock farms. Nevertheless, this is the largest OneHealth surveillance study in the Netherlands and a baseline study for future surveillance.

Of the four MRSA-CC398 groups, group D was the largest group, containing 65.2% of all isolates. The four groups differed mainly in the distribution of resistance genes and virulence genes; e.g. isolates in group A nearly all carried *erm*(A) (MLSB resistance), *ant(9)-Ia* (spectinomycin resistance)*, vga*(E) (pleuromutilins, lincosamide and streptogramin A resistance), and *dfrG* (trimethoprim resistance). Group B and D isolates differed in the prevalence of the trimethoprim resistance genes: group B isolates predominantly carried *dfrG*, while group D isolates often carried *dfrK*. There were also differences seen in the dissemination of the PVL genes *lukS-PV* and *lukF-PV*. All but one of the isolates carrying these virulence genes belonged to cluster C, and no PVL-positive isolates were from animal-related isolates. This is in line with other publications describing a lack of PVL genes in LA-MRSA-CC398 isolates with an epidemiological link to livestock (Stegger et al., 2012).

Within group D there was one branch containing predominantly MRSA-CC398 isolates originating from veal calves and these isolates often carried resistance genes conferring resistance to aminoglycosides and MLS_B_ [*aac(6′)-Ie- aph(2″)-Ia*, *aadD1* and *erm*(T)].

Interestingly, one branch within group C comprised MRSA-CC398 (*n* = 129) exclusively isolated from humans, and these isolates belonged to ST1232 and single or double locus variants thereof, and nearly always carried human-related virulence genes like PVL-, *sak*- and *scn*, and had SCC*mec* type V(5C2). In addition, these strains were often carriers of MLS_B_ and spectinomycin resistance genes [*erm*(A) variant and *ant(9)-Ia*] and lacked the tetracycline resistance gene *tet*(M). Previously it was shown that MRSA-CC398 isolates can be separated into two distinct phylogenetic groups: a livestock clade, termed CC398-IIa and a human clade, referred to as CC398-I/II-GOI. The human clade can be subdivided into two linages, L1 and L2. L1 usually carries the *SCCmec* type Vb (5C2&5) while L2 carries *SCCmec* type V (5C2) (Moller et al., 2019). The *scn* gene is strongly associated with the human MRSA-CC398 clade, while *tet*(M) is associated with the livestock clade (Stegger et al., 2012). This corroborates with our findings. Detection of the *erm*(A) variant encoding MLS_B_ resistance carried by ST1232 isolates can be challenging as it contains multiple point mutations compared to the reference sequence in ResFinder (Brodikova et al., 2025).

The isolates from the human lineage in group C therefore most likely belong to the CC398-I/II L2 clade as they were PVL- and *scn* positive, *tet*(M) negative and carried *SCCmec* type V (5C2). Only one PVL-positive isolate was found outside this group. This human isolate belonged to group D, was from ST398, carried *sak* and *scn*, and lacked *tet*(K), t*et*(L), and *tet*(M). PVL-positive MRSA-CC398 are endemic in countries like China and Vietnam, and MRSA-CC398 of ST1232 have previously been reported from other countries including Denmark, Czech Republic, Korea, Japan, Australia and New Zealand (Williamson et al., 2014; Moller et al., 2019; Nakaminami et al., 2020; Coombs et al., 2022; Kaneko et al., 2022; Brodikova et al., 2025). In 2023, 32% of diagnostic MRSA-CC398 isolates from the Dutch national MRSA surveillance were PVL-positive, which was higher than in previous years (de Greeff, 2024). The isolates from the human lineage in the current study were more often cultured from infections than those outside this branch pointing out their virulence. Persons colonized or infected by PVL-positive MRSA-CC398 had virtually no livestock contact. In Denmark, nearly all case-patients colonized or infected with PVL-positive MRSA-CC398 strains of the human variant had links to countries in mainland Asia (Moller et al., 2019). In the present study, only five out of 129 patients from the human ST1232 lineage had a known link with Asia (Vietnam *n* = 4; Thailand *n* = 1), indicating that this lineage seems to be spreading within the Dutch community, which is worrisome for a country with a low endemic level of MRSA. Recently, an outbreak with the human-adapted MRSA-CC398 lineage was detected in the Netherlands and 10 cases could be linked to a Thai massage salon. One of three employees carried the same MRSA lineage (van Schelven et al., 2024). More outbreaks like these might occur in the community without being detected. The questionnaire of the national human MRSA surveillance includes questions on recent travel history and hospitalization abroad, but does not detect links to Asia which are not travel-related. In addition, human-to-human transmission could occur more often with human-adapted MRSA-CC398 lineages compared to other MRSA-CC398 lineages, and the index case might not always be identified, complicating the identification of risk factors.

Remarkably, about one third of the persons outside this human MRSA-CC398 lineage also did not report any livestock contact, although (professional) contact with livestock is regarded the most important risk factor for MRSA-CC398 carriage. The Dutch Search and Destroy policy is based on active screening of high-risk groups (a.o. persons with professional livestock contact) upon hospital admission, preventive isolation, and treatment of MRSA carriers. Our results show that in the future it could get more difficult to clearly identify new risk groups carrying MRSA-CC398. Focusing on persons with professional livestock contact and persons that have been hospitalized in foreign countries will be insufficient to identify MRSA-CC398 linages such as the human-adapted ST1232, especially if they are community-acquired. The emergence of this human-adapted MRSA-CC398 lineage is a public health concern due to the potential higher disease burden and propensity to cause outbreaks. As significant differences in molecular characteristics exist between human-adapted MRSA-CC398 and livestock-associated MRSA-CC398, these might be used to differentiate these lineages. For future surveillance it should be realized that not all MRSA-CC398 are livestock-associated anymore. Currently, livestock is still an important source for MRSA-CC398 carriage in the Netherlands, but no control/eradication strategy is in place at national level in order to reduce the prevalence of MRSA in livestock. Continued WGS-based monitoring and surveillance of MRSA from a One Health perspective is necessary to identify emerging lineages in animals and humans. Genomic surveillance enables early detection to guide control measures and may help to limit spread of MRSA.

## Data Availability

The datasets presented in this study can be found in online repositories. The names of the repository/repositories and accession number(s) can be found in the article/Supplementary material.

## Footnotes

1
https://github.com/ncbi/amr

2
https://bitbucket.org/genomicepidemiology

## Members of the Dutch MRSA Surveillance Study Group

A. L. E. van Arkel, ADRZ medisch centrum, Department of Medical Microbiology, Goes; M. A. Leversteijn-van Hall, Alrijne Hospital, Department of Medical Microbiology, Leiden; W. van den Bijllaardt, Amphia Hospital, Microvida Laboratory for Microbiology, Breda; R. van Mansfeld, Amsterdam UMC, Vrije Universiteit Amsterdam, Department of Medical Microbiology and Infection Prevention, Amsterdam; K. van Dijk, Amsterdam UMC - location Vumc, Department of Medical Microbiology and Infection Control, Amsterdam; B. Zwart, Atalmedial, Department of Medical Microbiology, Amsterdam; B. M. W. Diederen, Bravis Hospital/ZorgSaam Hospital Zeeuws-Vlaanderen, Department of Medical Microbiology, Roosendaal/Terneuzen; H. Berkhout, Canisius Wilhelmina Hospital, Department of Medical Microbiology and Infectious Diseases, Nijmegen; A. Ott, Certe, Department of Medical Microbiology, Groningen; K. Waar, Certe, Department of Medical Microbiology, Groningen; W. Ang, Comicro, Department of Medical Microbiology, Hoorn; J. da Silva, Deventer Hospital, Department of Medical Microbiology, Deventer; A. L. M. Vlek, Diakonessenhuis Utrecht, Department of Medical Microbiology and Immunology, Utrecht; J. J. J. M. Stohr, Elisabeth-TweeSteden (ETZ) Hospital, Microvida laboratory for Microbiology, Tilburg; L. G. M. Bode, Erasmus University Medical Center, Department of Medical Microbiology and Infectious Diseases, Rotterdam; A. Jansz, Eurofins PAMM, Department of Medical Microbiology, Veldhoven; S. Paltansing, Franciscus Gasthuis & Vlietland, Department of Medical Microbiology and Infection Control, Rotterdam; A. J. van Griethuysen, Gelderse Vallei Hospital, Department of Medical Microbiology, Ede; J. R. Lo Ten Foe, Gelre Hospital, Department of Medical Microbiology and Infection Control, Apeldoorn; M. J. C. A. van Trijp, Groene Hart Ziekenhuis, Department of Medical Microbiology and Infection Prevention, Gouda; M. Wong, Haga Hospital, Department of Medical Microbiology, ‘s-Gravenhage; A. E. Muller, HMC Westeinde Hospital, Department of Medical Microbiology, ‘s-Gravenhage; M. P. M. van der Linden, IJsselland hospital, Department of Medical Microbiology, Capelle a/d IJssel; M. van Rijn, Ikazia Hospital, Department of Medical Microbiology, Rotterdam; S. B. Debast, Isala Hospital, Laboratory of Medical Microbiology and Infectious Diseases, Zwolle; E. Kolwijck, Jeroen Bosch Hospital, Department of Medical Microbiology and Infection Control, ‘s-Hertogenbosch; N. Al Naiemi, LabMicTA, Regional Laboratory of Microbiology Twente Achterhoek, Hengelo; T. Schulin, Laurentius Hospital, Department of Medical Microbiology, Roermond; S. Dinant, Maasstad Hospital, Department of Medical Microbiology, Rotterdam; S. P. van Mens, Maastricht University Medical Centre, Department of Medical Microbiology, Infectious Diseases & Infection Prevention, Maastricht; D. C. Melles, Meander Medical Center, Department of Medical Microbiology, Amersfoort; J. W. T. Cohen Stuart, Noordwest Ziekenhuisgroep, Department of Medical Microbiology, Alkmaar; P. Gruteke, OLVG Lab BV, Department of Medical Microbiology, Amsterdam; A. P. van Dam, Amsterdam Health Service, Public Health Laboratory, Amsterdam; J. Rahamat-Langendoen, Radboud University Medical Center, Department of Medical Microbiology, Nijmegen; J. van Prehn, Leiden University Center for Infectious Diseases, Department of Medical Microbiology and Infection Control, Leiden; B. Maraha, Regional Laboratory for Microbiology, Department of Medical Microbiology, Dordrecht; J. C. Sinnige, Regional Laboratory of Public Health, Department of Medical Microbiology, Haarlem; E. van der Vorm, Reinier de Graaf Groep, Department of Medical Microbiology, Delft; M. P. A. van Meer, Rijnstate Hospital, Laboratory for Medical Microbiology and Immunology, Velp; M. de Graaf, Saltro Diagnostic Centre, Department of Medical Microbiology, Utrecht; E. de Jong, Slingeland Hospital, Department of Medical Microbiology, Doetinchem; S. J. Vainio, St Antonius Hospital, Department of Medical Microbiology and Immunology, Nieuwegein; E. Heikens, St Jansdal Hospital, Department of Medical Microbiology, Harderwijk; M. den Reijer, Star-shl diagnostic centre, Department of Medical Microbiology, Rotterdam; J. W. Dorigo-Zetsma, TergooiMC, Central Bacteriology and Serology Laboratory, Hilversum; A. Troelstra, University Medical Center Utrecht, Department of Medical Microbiology, Utrecht; E. Bathoorn, University of Groningen, Department of Medical Microbiology, Groningen; J. de Vries, VieCuri Medical Center, Department of Medical Microbiology, Venlo; D. W. van Dam, Zuyderland Medical Centre, Department of Medical Microbiology and Infection Control, Sittard-Geleen; E. I. G. B. de Brauwer, Zuyderland Medical Centre, Department of Medical Microbiology and Infection Control, Heerlen; R. Steingrover, St. Maarten Laboratory Services, Department of Medical Microbiology, Cay Hill (St. Maarten); C. Oliveira dos Santos, Analytical Diagnostic Center N. V. Curaçao, Department of Medical Microbiology, Willemstad (Curaçao); F. Koene, Medical Laboratory Services, Department of Clinical Microbiology, Willemstad (Curaçao).
